# Geohash-Based High-Definition Map Provisioning System Using Smart RSU

**DOI:** 10.3390/s25175509

**Published:** 2025-09-04

**Authors:** Wangyu Park, Jimin Lee, Changjoo Moon

**Affiliations:** Department of Smart Vehicle Engineering, Konkuk University, Seoul 05029, Republic of Korea; wqpark123@konkuk.ac.kr (W.P.); easymean0417@konkuk.ac.kr (J.L.)

**Keywords:** HD map, Geohash, Smart RSU (Road Side Unit), PostgreSQL 15, PostGIS

## Abstract

High-definition (HD) maps are essential for safe and reliable autonomous driving, but their growing size and the need for real-time updates pose significant challenges for in-vehicle storage and communication efficiency. This study proposes a lightweight and scalable HD map provisioning system based on Geohash spatial indexing and Smart Roadside Units (Smart RSUs). The system divides HD map data into Geohash-based spatial blocks and enables vehicles to request only the map segments corresponding to their current location, reducing storage burden and communication load. To validate the system’s effectiveness, we constructed a simulation environment where multiple vehicle clients simultaneously request map data from a Smart RSU. Experimental results showed that the proposed Geohash-based approach achieved an average response time (RTT) of 1244.82 ms—approximately 296.3% faster than the conventional GPS-based spatial query method—and improved database query performance by 1072.6%. Additionally, we demonstrate the system’s scalability by adjusting Geohash levels according to road density, using finer blocks in urban areas and coarser blocks in rural areas. The hierarchical nature of Geohash also enables consistent integration of blocks with different resolutions. These results confirm that the proposed method provides an efficient and real-time HD map delivery framework suitable for dynamic and dense traffic environments.

## 1. Introduction

With the recent push toward the commercialization of autonomous driving, competition in autonomous driving technologies has become more advanced than ever before. Consequently, the types of external environmental information that vehicles must perceive and process have grown increasingly complex, requiring centimeter-level precision spatial data such as lane widths, road boundaries, intersection structures, and traffic signs. Although sensors such as LiDAR, cameras, and GPS can acquire this information in real-time, their performance deteriorates under adverse conditions such as inclement weather, changes in lighting, or sensor blind spots. To overcome these limitations, prior knowledge in the form of high-precision spatial data—such as road structure and traffic regulations—is required, and high-definition (HD) maps have emerged as a critical complement to real-time sensor data [1].

HD maps contain a variety of road objects, including lanes, traffic lights, signs, road boundaries, and roadside structures. Recently, even 3D point cloud-based terrain information has been utilized to enrich these maps [2]. Such high-precision spatial data play a crucial role in the perception-decision-control pipeline of autonomous driving systems. However, in practice, HD maps for autonomous vehicles can occupy tens to hundreds of megabytes per kilometer, and when high-resolution point clouds are included, the size can reach several gigabytes. This poses a burden on the storage capacity of the vehicle [2].

To address this issue, instead of storing the entire HD map onboard, a selective provisioning strategy has been considered, where only the data relevant to the vehicle’s current location is provided in real time by a central C-ITS (Cooperative Intelligent Transport Systems) server. This method involves dividing HD map data into segments based on road objects (links), and providing only the map information for the link corresponding to the vehicle’s current GPS location [3].

However, segmenting HD maps solely by road objects presents several limitations. First, road object units are not spatially continuous. While they are useful for expressing logical connectivity in road networks, spatially adjacent regions are often stored as separate links, which can fragment the map [3]. Second, road object units are not directly linked to the vehicle’s GPS coordinates. Since vehicle positions are typically represented using latitude and longitude, they do not directly map to road objects [4]. As a result, spatial computations and queries are required to identify the corresponding segment of the HD map, increasing system load and latency—especially detrimental for real-time communication with moving vehicles.

To overcome these limitations, this study proposes an HD map provisioning system based on Geohash spatial indexing. Geohash encodes latitude and longitude coordinates into fixed-length strings, dividing geographic space into uniform grid blocks [5]. This approach addresses the limitations of the conventional road object-based segmentation in the following ways. First, by leveraging Geohash’s continuous block-based indexing, the system can ensure spatial continuity of map data, even when adjacent roads are divided into different links. Since neighboring Geohash blocks share common prefixes, continuous data delivery is possible as the vehicle moves across regions. Second, Geohash enables direct mapping between a vehicle’s GPS coordinates and pre-indexed HD map blocks, eliminating the need for complex spatial queries. This reduction in computational overhead enhances real-time performance and contributes to improved safety and reliability in autonomous driving.

A real-time HD map provisioning infrastructure is also essential for this system. Conventionally, infrastructure components such as RSUs (Roadside Units), traffic signal controllers, CCTV, and VDS (Video Detection Systems) are integrated with a centralized C-ITS server to deliver data to vehicles. However, C-ITS centers are typically located far from the vehicles, introducing network latency. Moreover, as HD maps can be hundreds of megabytes in size, providing such data to a growing number of vehicles in a short period puts significant strain on the centralized servers [6].

Therefore, this study introduces Smart Roadside Units (Smart RSUs) as the primary map providers. Unlike traditional RSUs, which serve merely as transmitters, Smart RSUs are equipped with edge computing capabilities, possessing local storage and processing power [7]. By storing Geohash-partitioned HD map data locally, Smart RSUs can respond to vehicle requests in real-time based on their GPS-derived Geohash code. Being physically closer to the vehicles also helps reduce communication delays. Additionally, regional deployment of Smart RSUs can mitigate load concentration issues inherent to centralized systems [8].

Therefore, this study proposes a high-definition (HD) map provisioning system based on Geohash spatial indexing using Smart RSUs. Actual HD map data for autonomous driving was preprocessed and divided in advance into Geohash block units, and a database was constructed to map the road objects contained within each block. When a vehicle sends a request to the Smart RSU using its GPS-derived Geohash code, the server responds with the corresponding HD map data for that block. This allows the vehicle to receive only the necessary map segments in real-time without needing to store the entire HD map locally. To evaluate the system’s performance, a scenario was constructed in which multiple vehicles simultaneously connect to the Smart RSU and request map data. Experimental results showed that the Geohash-based method achieved an average response delay of 1244.82 ms, representing approximately a 296.3% improvement over the GPS-based method (4654.93 ms). In addition, the average database query time was measured at 15.90 ms, which is approximately 1072.6% faster than the GPS-based method (324.91 ms). These results demonstrate that the proposed method significantly improves the real-time performance and efficiency of HD map provisioning compared to traditional approaches.

The remainder of this paper is structured as follows: Section 2 reviews related work. Section 3 describes the system architecture and implementation of each module. Section 4 details the acquisition and preprocessing of HD map data. Section 5 presents experimental scenarios and performance evaluations. Finally, Section 6 discusses conclusions and future research directions.

## 2. Related Works

In the past, data processing in autonomous driving and intelligent transportation systems was primarily carried out either on the vehicle’s onboard computer or at centralized cloud servers [9]. However, as the volume of sensor data generated by vehicles has significantly increased, the computational resources of onboard systems have become insufficient for real-time processing. At the same time, transmitting all data to cloud servers has led to network latency and communication bottlenecks. To overcome these limitations, Roadside Units (RSUs) have been introduced. Evolving beyond simple communication relays, RSUs have developed into edge computing nodes capable of processing data near the vehicle. This enables partial offloading of computational tasks from the vehicle to RSUs, thereby reducing onboard processing burden, minimizing cloud dependency, and satisfying real-time operational requirements.

Liu et al. proposed an RSU-based computation offloading architecture in which the RSU processes tasks received from vehicles, demonstrating experimentally that this structure can reduce latency and improve execution success rates [10]. While prior RSU-based offloading studies have shown effectiveness in latency reduction and improved task reliability, they have limitations in handling large-scale data segmentation, provisioning, and real-time delivery. Cho et al. proposed an energy-efficient offloading technique that utilizes multiple RSUs deployed along a vehicle’s path as cooperative edge computing nodes [11]. By dividing computational tasks across multiple RSUs and having each node pre-compute results before the vehicle enters its communication range, their approach minimized total energy consumption and addressed the inefficiency of relying on a single RSU during high-speed driving. However, as their work primarily focused on energy optimization, it lacked comprehensive analysis regarding real-time performance factors such as latency and communication stability. Furthermore, the study did not adequately consider resource management and data synchronization between RSUs.

Thukkani et al. leveraged the hierarchical block structure of Geohash to aggregate vehicle location data at the regional level and combined this approach with a Redis-based distributed in-memory database to significantly enhance the performance of range queries and neighboring vehicle searches [12]. Their experiments showed that, compared to traditional RDBMS-based approaches, the proposed system achieved superior performance in low-latency location queries and handling large-scale concurrent connections. However, their work utilized Geohash merely as an indexing key for location lookup and did not explore the structural partitioning or management of large-scale spatial databased on Geohash.

To address these limitations in existing research, this paper proposes a novel approach that utilizes RSUs not only as computation offloading nodes but also as the core entities responsible for provisioning HD map data. In addition, Geohash is employed not just as a lookup optimization tool but as the fundamental unit for HD map request handling and data management. While previous RSU studies focused on offloading and energy efficiency, the proposed system uniquely integrates RSU and Geohash technologies to construct a unified architecture that supports the partitioning, management, and provisioning of high-definition spatial data. This design improves both the real-time responsiveness and efficiency of HD map delivery systems, offering a distinctive contribution beyond the scope of earlier research.

## 3. System Architecture and Implementation

This section describes the architecture of the vehicle client and Smart RSU server, as well as the implementation of their communication structure.

### 3.1. System Architecture

**Figure 1:**
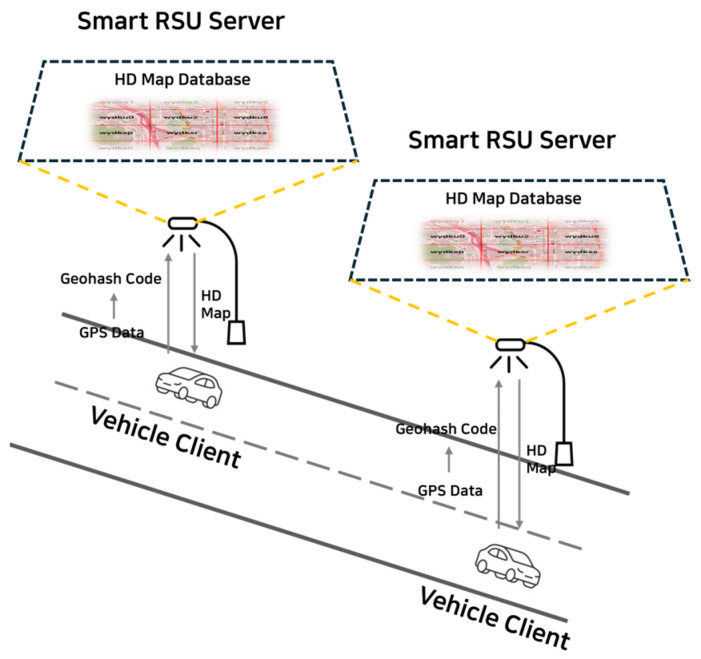
System Architecture.

**Figure 2:**
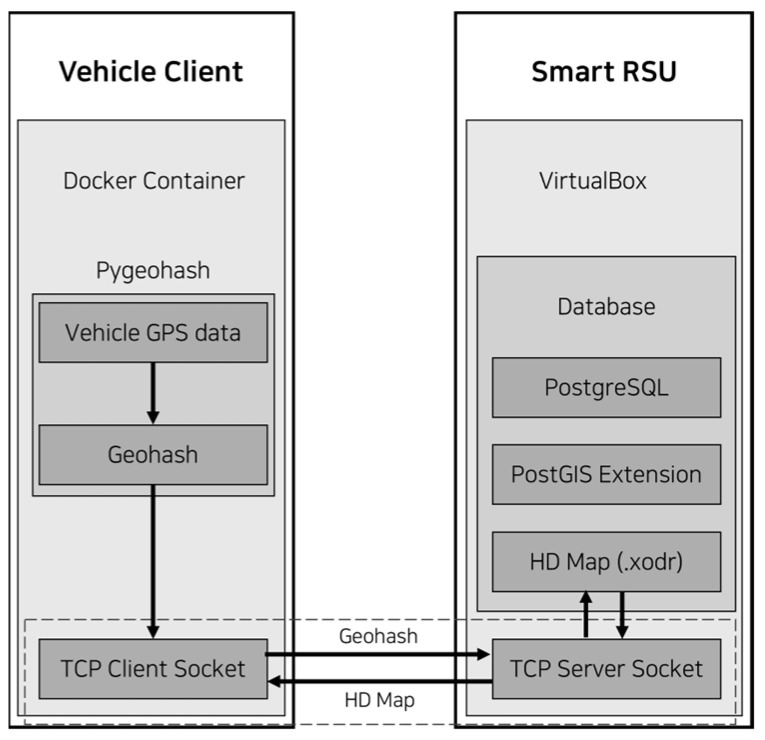
Implementation Structure and Data Flow.

Figure 1 illustrates the overall system architecture proposed in this study, which consists of two main components: the vehicle client and the Smart RSU server. The vehicle client requests high-definition (HD) map databased on its current location and utilizes the received map data to support driving-related tasks. The Smart RSU server is responsible for providing the corresponding HD map data to the vehicle client based on the requested location. Figure 2 explains the actual implementation structure and the flow of data within the system.

### 3.2. Vehicle Client

The vehicle client is a module that virtually simulates a real autonomous vehicle and is responsible for requesting HD map data from the Smart RSU and receiving the corresponding response. As shown in Figure 2, the vehicle client was implemented in a Docker-based container environment [13]. Docker 28.1.1 was chosen for its lightweight virtualization, which enables easy deployment and execution, as well as the ability to independently generate multiple vehicle clients—making it well-suited for large-scale experimental setups. The overall experimental environment was configured on Windows 11.

**Figure 3:**
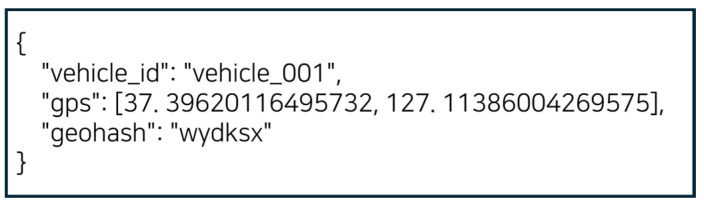
JSON-formatted request message from the vehicle client.

The core function of the vehicle client is to request HD map data for the area corresponding to the vehicle’s current location (GPS coordinates). First, the vehicle client assigns arbitrary latitude and longitude values to simulate GPS data. This GPS data is then encoded into a Geohash string using the Python 3.10 APIlibrary pygeohash [14], which is referred to as the Geohash code. The vehicle client sends this Geohash code to the Smart RSU to request the HD map data corresponding to that region. The request is transmitted via a TCP communication method using a TCP client socket, which delivers the request message—containing the Geohash code—to the Smart RSU server [15]. The message includes the vehicle identifier (vehicle ID), latitude and longitude coordinates, and the Geohash code. As shown in Figure 3, the message is formatted in JSON.

After sending the request, the vehicle client receives the .xodr file corresponding to the requested Geohash code from the Smart RSU server and saves it in the local directory. The .xodr file is a standardized HD map format designed for autonomous driving applications [16]. When using the Geohash block format, map data can be spatially divided more efficiently, allowing the vehicle to selectively receive only the necessary areas. This reduces data transmission volume and improves processing speed. Additionally, it enables rapid switching between adjacent blocks during continuous driving, which is advantageous for ensuring real-time responsiveness.

### 3.3. Smart RSU

The Smart RSU server is responsible for providing the HD map data corresponding to the Geohash block based on the Geohash code received from the vehicle client. To simulate a realistic autonomous driving environment, the Smart RSU server was implemented on a VirtualBox-based virtual machine running Ubuntu 24.04.2, as shown in Figure 2, and was composed of the components listed in Table 1.

This environment was selected because VirtualBox 7.1.8 allows for independent network configuration and resource allocation, separate from the Windows-based local PC environment used for vehicle client development. In addition, the network was configured in bridged mode, allowing the Smart RSU server to be assigned a unique IP address independent of the host system [17]. This setup effectively emulates the operational characteristics of a standalone Smart RSU device in real-world deployment.

Furthermore, the service coverage of the Smart RSU was defined within a practical distance range that satisfies the target V2I communication quality. According to standards and field measurement reports, typical coverage spans 300–500 m in dense urban areas, 500–700 m in suburban corridors, and up to 0.8–1.0 km on highways. These ranges were adopted as default reference values, acknowledging potential variations depending on the wireless technology employed (ITS-G5/IEEE 802.11p, C-V2X/NR-V2X) and the surrounding urban environment.

Road geometry and traffic density are critical factors influencing RSU coverage. In areas with short intersection spacing, concentrated turning or branching points, and high traffic density during peak hours, higher request frequency necessitates smaller service ranges and denser RSU deployment. In contrast, larger coverage can be supported in suburban and highway corridors with lower density and longer straight segments. Based on these considerations, the RSU map provision unit was set to approximately 1 km × 1 km, and the effective service range of the RSU was assumed to ensure continuity within this scale.

The primary function of the Smart RSU server is to provide HD map data in .xodr format for the region corresponding to the Geohash code received from the vehicle client. The vehicle’s TCP client socket sends a request message containing the Geohash code, which is received by the TCP server socket of the Smart RSU. The server uses this Geohash code as a key to query the PostgreSQL database for the file path of the corresponding .xodr file. Once the file path is retrieved, the server accesses the pre-stored .xodr file (in OpenDRIVE format) from the local file system and transmits it to the vehicle client via the TCP server socket. The vehicle client then saves the received file locally under the name received_hdmap.xodr for further use.

## 4. Acquisition and Preprocessing of High-Definition Map Data

### 4.1. Structure of High-Definition Map Data

In this study, to construct an HD map provisioning system suitable for use in real-world autonomous driving environments, we utilized a combination of road structure data and visual feature data provided by Naver Labs [18]. The road structure data includes essential information that forms the basis of the driving path, such as lane geometry, road connectivity, intersections, and the locations of traffic signs. The detailed components of this dataset are summarized in Table 2.

Conventional centralized high-definition (HD) map provisioning services based on the C-ITS center face limitations in delivering HD maps in real time due to bottlenecks caused by the large volume of map data. Therefore, this study aims to demonstrate that the Smart RSU-based approach can ensure real-time performance even as the size of HD map data increases. While HD map data is typically structured around spatial geometry, this study additionally incorporates visual attribute data corresponding to each road object in order to further increase data volume and more reliably evaluate the system’s real-time processing limits. The visual attribute data provides semantic information required for sensor perception, such as the type and orientation of traffic signs, the pattern of stop lines and crosswalks, and the color and width of lane markings. These data are stored in .h5 format, containing visual features extracted through deep learning-based perception algorithms. Each .h5 file is organized by road object ID, which corresponds to link or lane objects in the HD map database [19].

### 4.2. Geohash-Based Spatial Segmentation and Mapping

**Figure 4:**
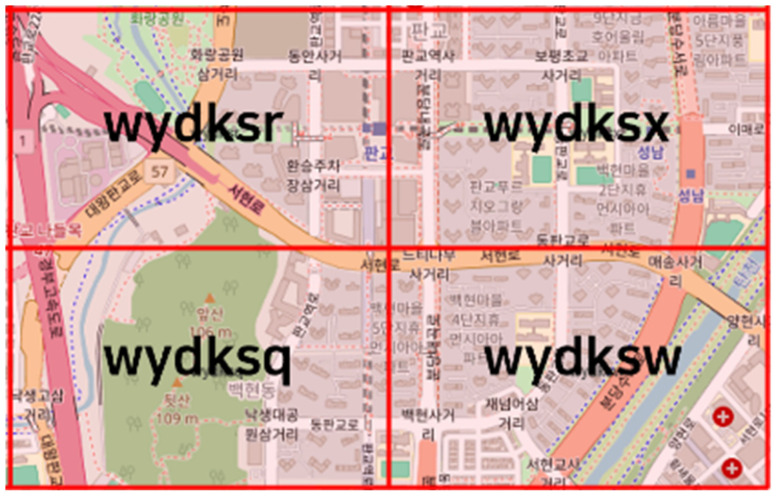
Visualization of Geohash level 6 blocks.

The Geohash segmentation level was determined based on several key considerations. If the block size is too large, it may include an excessive amount of HD map data, increasing communication overhead and processing time. Conversely, if the block is too small, communication may occur too frequently, negatively impacting network throughput and latency. An analysis of HD map data for the Pangyo region in South Korea using QGIS [20] confirmed that a single Geohash level 6 block can individually encompass major intersections and road segments in urban areas, as shown in Figure 4. This allows vehicles to request and process only the map data required for short-range driving paths. In addition, as long as the vehicle does not move a significant distance, it can continue driving within the same Geohash region, thereby reducing unnecessary map requests and alleviating the overall processing load on the system. Furthermore, the average size of a map file contained within a single Geohash block was approximately 230 MB, which is a practical size for storage within an autonomous vehicle’s onboard system and demonstrates the feasibility of lightweight, block-based map delivery. In addition, the map file size contained in a single Geohash block is approximately 230 MB on average. This corresponds to the context introduced in the Introduction, where real-world HD maps are reported to require tens to hundreds of megabytes per kilometer, and reflects the adoption of a 1 km spatial block scale. Accordingly, the evaluation of the block-based delivery mechanism was conducted under realistic conditions. The measured values in this study (RTT, database lookup time, and per-request payload) thus represent a practical operating range, demonstrating that the data size is feasible for in-vehicle systems and further substantiating the effectiveness of lightweight, block-level map delivery.

In an additional comparison with 50 vehicles, Level 6 exhibited an average RTT of 1.6 s with a block size of approximately 230 MB, whereas Level 5 resulted in a block size of about 700 MB with an RTT of 3 s, and Level 4 in a block size of about 2.5 GB with an RTT of 6 s. These results indicate that larger block sizes lead to increased transmission delays, confirming that Level 6 provides the most practical balance between block size and latency.

Conventional approaches that retrieve map databased on individual road links often involve repeated searches and computations across multiple links to obtain the map for a specific region. This process increases computational overhead and leads to query processing delays. In contrast, using the Geohash block method eliminates the need for complex spatial operations by utilizing predefined string-based keys. This allows all road links within a specific block to be retrieved at once. As a result, the management of map data is simplified from a link-based to a block-based approach, significantly improving storage and retrieval efficiency. Therefore, the Geohash technique provides an advantage in terms of real-time performance by addressing the computational burden and inefficient data management issues inherent in traditional link-based query methods.

The HD map data was stored in a spatial database using PostgreSQL 15 with PostGIS support [21]. The database was structured as shown in Table 3. All tables include a geom field that represents the spatial geometry of each object, such as points, lines, or polygons, expressed in coordinate form. From this geometry field, the geographic center point (latitude and longitude) of each object is calculated. Based on this central point, a Geohash level 6 code is generated and stored in the geohash6 field. This field serves as the identifier for determining which spatial block the vehicle client’s request corresponds to.

For example, if the geometry information of a linkid object yields a centroid at latitude 37.4° and longitude 127.1°, the Geohash level 6 encoding would produce the code wydku3, which is then stored in the geohash6 column. This process is performed once during the preprocessing phase. The Smart RSU server then uses the geohash6 field to query and retrieve HD map data corresponding to the requested region. The assignment of Geohash codes was implemented using spatial operations within the database and the external Python 3.10 library pygeohash.

Based on the high-definition map data annotated with Geohash codes as shown in the Table 4 above, pre-generated .xodr files were created for each Geohash code. The .xodr file is a high-definition map file formatted according to the OpenDRIVE standard and contains detailed road object information required for autonomous driving, such as road geometry, lane information, and traffic signs. When this file is delivered to the vehicle client, the client can utilize the detailed road environment information for path planning and driving decision-making.

To generate the .xodr files, spatial objects such as road centerlines, lanes, nodes, stop lines, and traffic signs were retrieved for each Geohash code and structured in JSON format. Then, using a Python 3.10-based conversion module (json_to_xodr.py), the data was converted into the OpenDRIVE schema by mapping elements such as roads, lanes, and junctions to XML nodes and constructing a hierarchical structure accordingly [16].

The converted .xodr files were stored in a single local repository, and since the storage unit and transmission unit coincide at the block level, this study did not introduce a hierarchical structure across different map elements. However, in large-scale operations where differential updates at the element level may be required, element-specific hierarchization would be a valid direction for future extensions.

**Figure 5:**
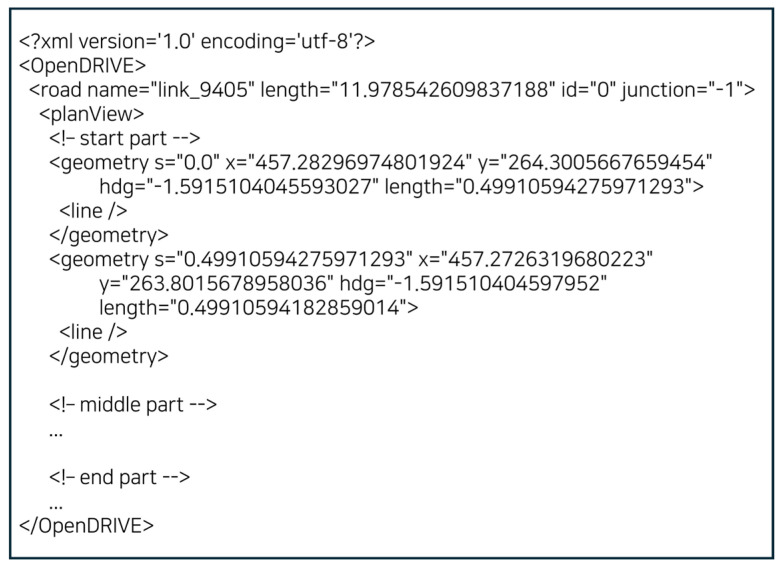
Internal Structure of a Generated OpenDRIVE (.xodr) File.

Figure 5 illustrates the internal structure of a generated OpenDRIVE (.xodr) file. The OpenDRIVE schema is hierarchically organized around the <road> node, which serves as the core unit encompassing each road object. The schema is primarily structured with the <planeView> and <lanes> nodes. The <road> node at the top level contains metadata such as a unique road identifier, road length, and junction presence. The <planeView> node at the lower level describes the continuous road geometry using lines, arcs, and splines, while the <lanes> node defines the detailed configuration of drivable lanes, lane boundaries, and center lanes. When necessary, the <junction> node specifies the connectivity between roads. The converted files are saved with filenames that include the corresponding Geohash code (e.g., hdmap_wydku3.xodr).

**Figure 6:**
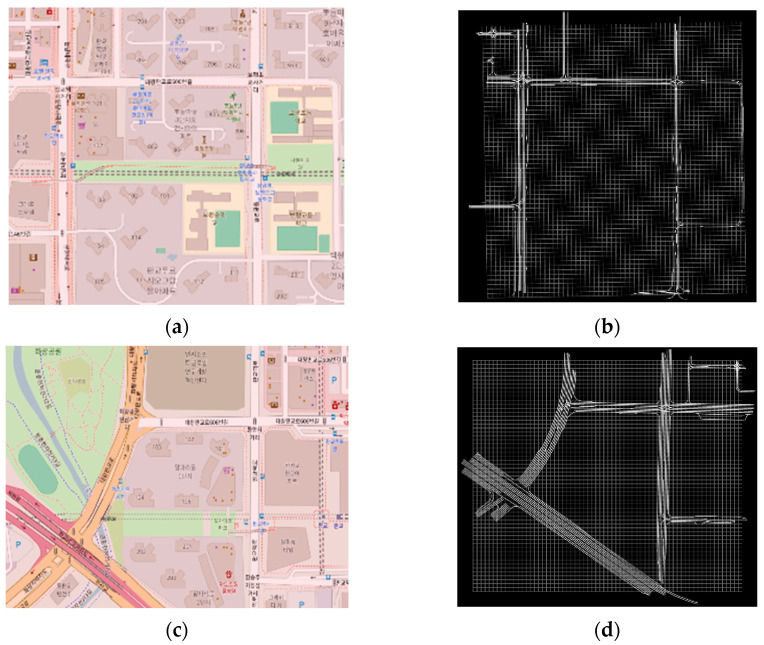
Actual map and visualization of .xodr file for wydksx, wydksr. (**a**) The actual map of the wydksx region, (**b**) Visualization of .xodr file for wydksx, (**c**) The actual map of the wydksr region, (**d**) Visualization of .xodr file for wydksr.

Figure 6 shows a visualization result by uploading the generated .xodr files to the Online OpenDRIVE Viewer, allowing comparison with the actual map [22]. It confirms that each file contains multiple links corresponding to the respective Geohash region.

This pre-generation process is intended to reduce the computational burden on the Smart RSU of converting large-scale spatial data in real time upon receiving a request from a vehicle client, thereby enabling immediate provision of high-definition map data for the requested Geohash region. 

## 5. Experimental Overview and Measurement of Evaluation Metrics 

### 5.1. Overview of the Experiments 

In this study, experiments were conducted to evaluate the efficiency of the Geohash-based spatial indexing method and the effectiveness of the HD map provisioning system using Smart RSUs (edge servers). The experiments were designed around four main comparative criteria. First, to compare delay times based on request methods, the response time was measured when the vehicle client requested map data using the Geohash-based method versus the traditional GPS radius-based method. Second, to compare performance based on the data provider, the processing speed and system load were evaluated when HD map data was provided by the Smart RSU versus by a centralized C-ITS cloud server(GCP, Google LLC, Seoul, Republic of Korea). Third, to assess the scalability of the Smart RSU server, system resource usage was monitored as the number of vehicle clients increased. Fourth, the accuracy and completeness of HD map data provided by the Smart RSU were verified to ensure no data loss occurred during transmission. In addition, the system’s scalability was evaluated by verifying the ability to flexibly adjust the size of spatial blocks according to road network complexity. All experiments were conducted in the same testbed location—Pangyo Intersection, South Korea—to ensure consistent conditions for performance analysis and evaluation.

### 5.2. Experiment 1: Comparison of Delay Time According to Request Method

The purpose of this experiment is to quantitatively compare the system response delay (RTT; Round Trip Time) and database processing time between the traditional GPS-coordinate-based request method and the proposed Geohash-code-based request method for retrieving high-definition map data.

The Geohash-based request experiment in this study was conducted using the wydksx block, which includes Pangyo Station Intersection in South Korea. This block corresponds to Geohash Level 6 (1.22 km × 0.61 km). For comparison, the GPS-based request experiment used the latitude and longitude of the center point of the wydksx block (37.394701, 127.116833), and retrieved road object data within a radius of 0.8627 km—chosen to match the area size of a Geohash Level 6 block.

The size of the received data was approximately 236.31 MB for the Geohash method and 245.37 MB for the GPS method, maintaining similar volumes. Each method was tested 100 times using the same geographic region.

First, the system response delay time was evaluated. This delay is defined as the total round-trip time from the moment the vehicle client sends a request containing the Geohash code to the Smart RSU server, to the moment the .xodr file is successfully transmitted back to the client. This includes network transmission time, server-side request processing time, file retrieval time, and file transfer time, thereby representing the comprehensive responsiveness of the system.

**Figure 7:**
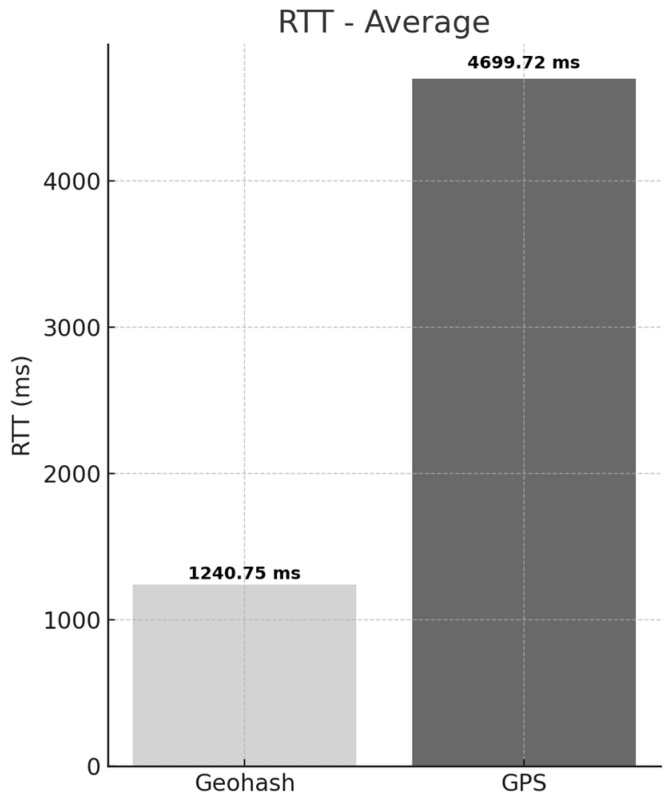
Comparison of Average RTT between Geohash-based and GPS-based Requests.

The experimental results are presented in Figure 7. The average RTT for the Geohash-based method was measured at 1240.75 ms, whereas the GPS-based method recorded an average RTT of 4699.72 ms. This indicates that the Geohash-based method achieves a response time approximately 278.8% faster than the GPS-based method. These findings suggest that the Geohash-based approach effectively overcomes structural limitations inherent in existing methods, which rely on real-time spatial computations such as PostGIS distance queries based on GPS coordinates.

In contrast, the Geohash method enables efficient data retrieval through simple string matching, significantly reducing computational and lookup overhead. This allows even large-scale spatial data such as high-definition maps to be provided in real time. As a result, vehicles can perform critical driving decisions—such as route changes, intersection entry, or emergency response—without delay, thereby enhancing driving safety and overall system reliability.

Moreover, the average RTT of 1240.75 ms (approximately 1.2 s) satisfies the map update interval requirements of 1 to 5 s presented in prior HD map research [9], and more specifically, meets the recommended update interval of under 1–2 s for urban driving environments. Therefore, the proposed Geohash-based method demonstrates sufficient performance to support real-time HD map provisioning for autonomous vehicle decision-making.

The next experiment compares the database processing time. This metric refers solely to the time required for the Smart RSU server to query the PostgreSQL 15 database using the received Geohash code and retrieve the corresponding file path of the .xodr map file. This time was measured independently to evaluate the performance difference stemming from the structural divergence in data lookup mechanisms between the two request methods.

**Figure 8:**
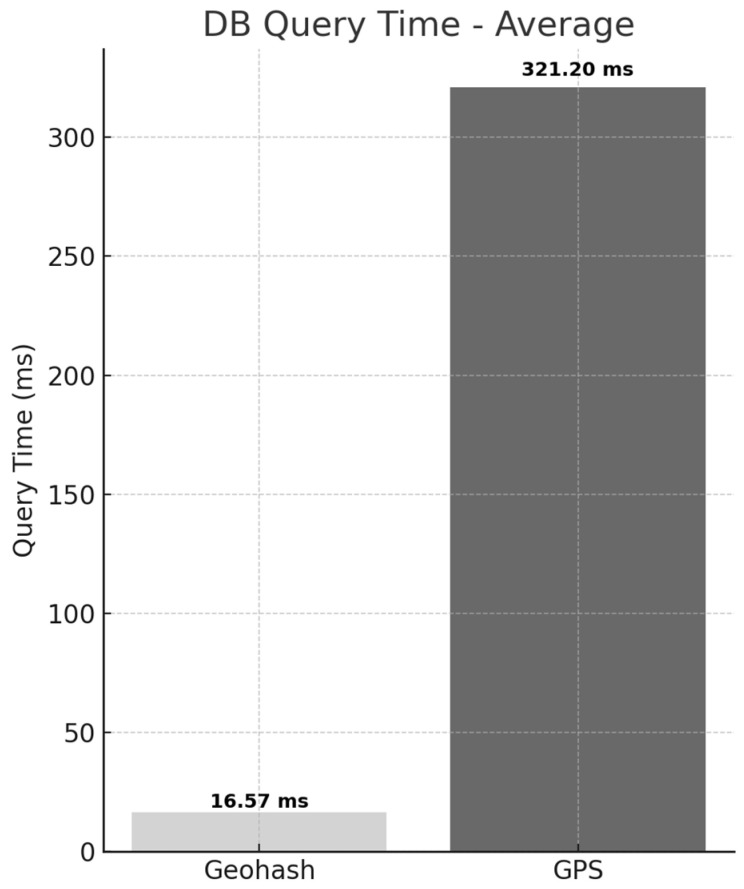
Comparison of Average Database Query Time between Geohash-based and GPS-based Requests.

The experimental results are shown in Figure 8. The average database query time for the Geohash-based method was 16.57 ms, whereas the GPS-based method recorded an average of 321.20 ms. This indicates that the database processing speed of the Geohash-based method is approximately 1838.4% faster than that of the GPS-based method. While the database processing speed showed a 1,838.4% improvement, the RTT improved by only 278.8%. This discrepancy is attributed to the structural characteristics of the GPS-based method. The GPS-based method performs real-time spatial queries to search for all relevant road objects, resulting in significantly longer database processing times. However, once the data is retrieved, it is transmitted in a relatively straightforward manner, allowing for faster response handling. In contrast, although the Geohash-based method enables faster data retrieval through lightweight indexing, it involves additional steps such as file lookup and transmission, which limit the overall improvement in RTT.

This significant performance gap stems from fundamental structural differences in the data retrieval processes of the two approaches. The Geohash method retrieves high-definition map data using a simple condition such as WHERE geohash6 = ’wydksx’, allowing direct access to the corresponding .xodr file path through a single string-matching operation. Since this approach avoids full table scans and leverages indexed lookups, it enables faster query execution while minimizing CPU resource usage.

In contrast, the GPS-based method involves spatial distance calculations between the vehicle’s latitude and longitude coordinates and surrounding road objects. This spatial computation introduces overhead, leading to substantially longer query times than the Geohash approach.

The reduced database processing delay in the Geohash method directly contributes to the lower overall system response time (RTT) observed in previous experiments. Therefore, the Geohash-based approach minimizes database query overhead and is well-suited for real-time high-definition map delivery systems, particularly in autonomous driving environments where continuous and consistent responses to frequent data requests are critical.

In addition, this study analyzed the scalability of the two approaches under concurrent vehicle requests. When 30, 50, and 100 vehicles simultaneously accessed the RSU to request HD map data, the GPS-based method exhibited severe performance degradation. The average RTT sharply increased from 5.9 s to more than 12.2 s as the number of concurrent requests grew, and the database query latency rose from 428.5 ms to 921.7 ms. This degradation is attributed to bottlenecks in PostGIS caused by repeated distance calculations, spatial index searches, and multi-table joins during the ST_DWithin operation. In contrast, the Geohash-based method showed only a moderate increase, from 1.3 s with 30 clients to 2.7 s with 100 clients, while the database query latency remained stable within the range of 20–35 ms.

These results demonstrate that the Geohash-based approach maintains stable performance even under high query concurrency, owing to its lightweight string comparison and pre-indexed mapping structure. Particularly in autonomous driving environments, where tens to hundreds of vehicles continuously generate real-time requests, the proposed Geohash-based method effectively mitigates the bottlenecks and performance degradation risks of PostGIS, thereby experimentally proving its suitability for real-time HD map provisioning systems.

### 5.3. Experiment 2: Performance Comparison Based on Map Data Provider

The purpose of this experiment is to quantitatively compare the impact of different high-definition map data providers—Smart RSU (Konkuk University, Seoul, Republic of Korea) and Cloud(Google LLC, Seoul, Republic of Korea)—on system response delay (RTT; Round Trip Time). In both environments, the Geohash-based request method was used to deliver data of identical size and structure, enabling a fair evaluation of performance differences arising solely from architectural disparities between the two providers.

The experiment was conducted using the wydksx Geohash block (Level 61.22 km × 0.61 km), which corresponds to the Pangyo Station intersection area in South Korea. The vehicle client sent the same Geohash code (wydksx) to both the Smart RSU and the Cloud server. Each server then transmitted the same pre-generated .xodr file (approximately 236 MB) in response.

For the cloud environment, a Google Cloud Platform (GCP, Google LLC, Seoul, Republic of Korea) VM instance was configured to serve as the C-ITS center, while the Smart RSU was implemented as an edge node operating on a VirtualBox-based environment. To simulate varying traffic loads, the experiment was repeated with 30, 50, and 100 vehicles making concurrent requests. The RTT for each vehicle was recorded until all requests were completed, and the average RTT per scenario was then calculated for comparison.

**Figure 9:**
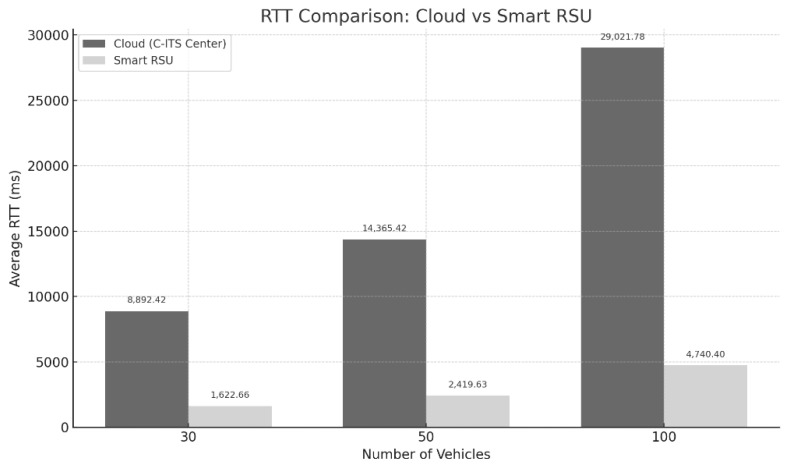
RTT Comparison Between Cloud and Smart RSU Under Varying Vehicle Loads.

As shown in Figure 9, the Smart RSU consistently recorded lower RTT values than the Cloud across all test scenarios. As the number of vehicles increased, the performance gap between the two environments widened, with the Cloud environment exhibiting a non-linear increase in RTT as the request load grew.

This result can be attributed to fundamental structural differences in the data provisioning scope and processing methods of each system. In this study, the Smart RSU was designed to manage only a single designated Geohash block (e.g., wydksx) and directly retrieve and transmit the corresponding .xodr file from its local file system. In contrast, the Cloud server is structured to handle concurrent requests from multiple regions (e.g., wydksx, wydksw wydksr, etc.), and the high-definition map data is distributed across different areas.

Moreover, the Cloud must perform spatial queries or coordinate-based dynamic lookups for each request, which, combined with the overhead introduced by virtualization layers and multiple network hops inherent to cloud infrastructure, results in significantly increased latency. Thus, the observed RTT differences are not merely due to differences in computing environments, but rather reflect deeper architectural distinctions in request handling and data retrieval strategies.

The performance comparison experiment confirms that the Smart RSU achieved response times 4 to 5 times faster than the Cloud (C-ITS Center) on average. Notably, this performance advantage became even more pronounced under higher vehicle loads. These results empirically demonstrate that the Smart RSU is more suitable than a centralized cloud-based architecture for serving high-definition map data in autonomous driving environments where real-time responsiveness is critical.

In this study, the experiments were conducted up to 30, 50, and 100 simultaneous vehicle requests, but the proposed system architecture is inherently scalable to larger traffic flows. Since Smart RSUs operate as distributed edge units, traffic requests are naturally partitioned by road segments, and in overload situations, requests can be further alleviated through load sharing among neighboring RSUs.

Moreover, the Geohash-based indexing scheme processes queries through simple string comparisons, ensuring that the computational cost increases linearly with the number of requests while minimizing CPU resource usage. In large-scale environments, resource management and scheduling mechanisms can also be incorporated. For example, round-robin request distribution, priority-based scheduling for safety-critical requests, and load balancing among adjacent RSUs can be seamlessly integrated into the modular design of the RSU server.

Therefore, although the experiments were limited to 100 vehicles, the distributed edge-based architecture and lightweight Geohash indexing structure confirm that the proposed system can be effectively applied to large-scale autonomous driving environments with significantly higher traffic volumes.

### 5.4. Experiment 3: Evaluation of RSU Server Resource Utilization

This experiment was conducted to evaluate the resource efficiency of the Smart RSU server as the number of vehicles increases. In a high-definition map provisioning system, the RSU must handle simultaneous requests from multiple vehicles, making it essential to quantitatively analyze the impact of increased vehicle traffic on the server’s computational and communication resources. In particular, since the Smart RSU is responsible for real-time data processing and transmission at the edge, CPU usage (indicating computational load) and network throughput (indicating communication load) serve as key indicators for assessing the system’s scalability and stability.

**Figure 10:**
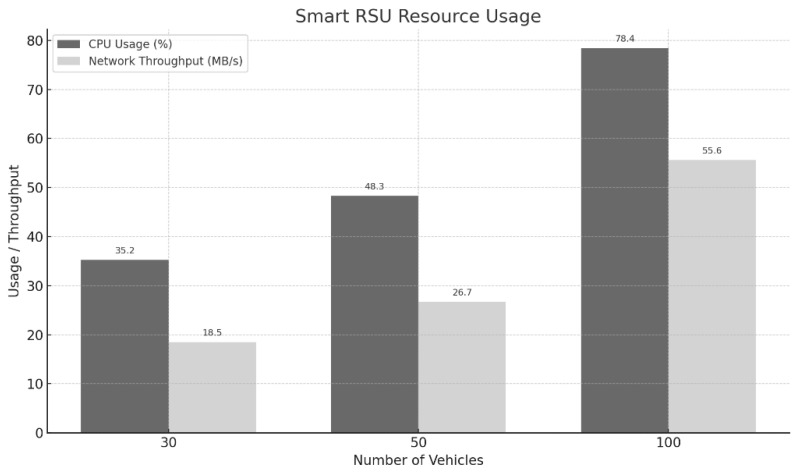
CPU Usage and Network Throughput of Smart RSU Under Increasing Vehicle Loads.

Figure 10 presents the average CPU usage (%) and average network throughput (MB/s) of the Smart RSU server as the number of vehicles increases from 30 to 50 and then to 100. The results show a clear upward trend in CPU usage, rising from 35.2% with 30 vehicles to 48.3% with 50 vehicles, and reaching 78.4% with 100 vehicles. This increase reflects the higher volume of simultaneous map retrieval and file transmission tasks that the RSU must handle as the number of vehicles grows. Similarly, network throughput also increased steadily—from 18.5 MB/s (30 vehicles) to 26.7 MB/s (50 vehicles), and 55.6 MB/s (100 vehicles)—as more .xodr files were required to be transmitted concurrently.

These results demonstrate that the Smart RSU server can handle up to 100 vehicles with a linear increase in resource usage, maintaining stable performance. The fact that CPU usage remained below 80% suggests that the server still has some processing headroom, even at higher loads. Additionally, the increase in network throughput occurred at a consistent rate without signs of congestion or overload. Therefore, the findings indicate that the Smart RSU server can reliably provide services even under high concurrency, and possesses sufficient scalability to support larger-scale deployments through potential hardware upgrades.

### 5.5. Experiment 4: Evaluation of Data Consistency and Scalability

The purpose of this experiment is to evaluate whether the high-definition maps provided by the Smart RSU to vehicle clients are delivered stably and continuously without any data loss. In particular, the experiment focused on verifying boundary continuity between adjacent blocks in a Geohash-based map provisioning structure where the data is divided and delivered in block units.

First, the number of boundary roads in four Geohash blocks—wydksx, wydksr, wydksq, and wydksw—was calculated. Then, for each pair of adjacent blocks, the presence of matching link IDs was examined to determine continuity. Continuity was defined as the ratio of boundary roads in the reference block that also exist in the neighboring block, where a higher ratio indicates a more seamless connection between the two blocks. The evaluation results are shown in Table 5, and all adjacent block pairs demonstrated 100% continuity.

These findings confirm that the boundary roads of the high-definition maps received by vehicles are consistently shared across Geohash blocks, enabling uninterrupted map reception along the vehicle’s driving path without any data omission.

In addition, Geohash offers scalability by enabling flexible adjustment of spatial block sizes based on its hierarchical encoding structure, which can adapt to varying road network complexities. For example, in dense urban areas such as the Pangyo intersection—where intersections are closely clustered and road objects are intricately distributed—Geohash level 6 segmentation is required for precise HD map management. In practice, a single level 6 block in the Pangyo region resulted in an average .xodr file size of approximately 230 MB.

In contrast, for regions with low road density and simpler structures—such as Daehwa-myeon in Pyeongchang-gun, Gangwon Province—the average file size of .xodr files generated with the same level 6 setting was approximately 60 MB. In such simple areas, using broader blocks (e.g., level 5 or higher levels) is more efficient, helping reduce storage requirements and communication overhead.

Moreover, since Geohash has a hierarchical structure in which higher-level codes contain lower-level ones, the system can be expanded without structural conflict. For example, even if the system was initially designed using level 6 blocks, it can later be extended to store outer suburban areas using level 5 blocks without any inconsistency or system error. This is because all blocks are managed coherently within the prefix-based Geohash key structure. Therefore, the proposed system ensures both architectural flexibility and scalability in HD map provisioning.

## 6. Conclusions and Future Work

This study proposed a system architecture for real-time high-definition (HD) map provision using a Smart RSU-based Geohash spatial indexing method and validated its performance using actual autonomous driving map data. To overcome the spatial computation overhead and communication delay issues inherent in conventional GPS-based radius query methods, the system adopted a lightweight indexing scheme based on Geohash strings to maximize data retrieval efficiency. Furthermore, the role of the map provider was shifted from a centralized cloud-based C-ITS server to an edge-based RSU, alleviating both response delay and server load.

Experimental results showed that the proposed Geohash-based request method achieved significant improvements in average response time (RTT) and database query processing time compared to the GPS-based method. In addition, the Smart RSU maintained consistent and stable response performance even as the number of vehicles increased, and its resource usage remained within acceptable limits, demonstrating the system’s scalability and stability. Notably, the Smart RSU architecture, which searches for and transmits pre-generated .xodr files by region, proved to be an effective solution for addressing real-time requirements and mitigating network bottlenecks in autonomous driving environments.

The continuity verification between adjacent Geohash blocks confirmed that no data loss or disconnection occurred at block boundaries, ensuring seamless map reception along vehicle routes. Furthermore, the scalability evaluation revealed that adjusting Geohash levels based on regional data complexity (e.g., urban vs. rural) can reduce storage and communication costs, confirming the flexibility of the system.

In summary, this study presents a practical architecture that achieves both structural efficiency and real-time performance in HD map provisioning. It holds significance in that it experimentally validates the potential of edge-based map provisioning technologies for the future expansion of large-scale autonomous driving infrastructure.

In future work, we plan to implement a distributed processing architecture among multiple Smart RSUs and develop a dynamic HD map update system, while conducting a comprehensive performance evaluation of real-time responsiveness and communication reliability in a C-V2X-based environment. Additionally, we aim to establish a methodology for flexibly adjusting Geohash levels based on regional road characteristics to achieve a balance between spatial efficiency and real-time performance.

Furthermore, while this study assumed Smart RSUs to be statically deployed, in real-world environments, mobile RSUs mounted on vehicles, buses, or drones can also play an important role. Accordingly, future work will investigate the applicability of mobile RSUs and evaluate how coverage changes and latency variations caused by mobility affect system performance, thereby further extending the generality and practicality of the proposed architecture.

## Figures and Tables

**Table 1:** System configuration for the Smart RSU server.

Item	Description
OS	Ubuntu 24.04.2 LTS (VirtualBox 7.1.8 VM)
DBMS	PostgreSQL 15, PostGIS Extension
Communication	Python 3.10 socket-based TCP server
Storage	Directory storing .xodr files by Geohash blocks
External Libraries	Psycopg2, Shapely, PyProj, PyGeohash 2.1.0

**Table 2:** Components of road structure data.

Category	File Name	Description	Format
Lane	Pangyo_A1_Lane_3D	Land information on the road	.shp, .dbf, .shx, .prj
Stop Line	Pangyo_A2_STOP_3D	Stop line location and geometry information	.shp, .dbf, .shx
Road Centerline	Pangyo_A3_LINK_3D	Road connectivity information between nodes	.shp, .dbf, .shx, .prj
Traffic Sign(Direction)	Pangyo_B2_SURFSIGN_DIRECTION_3D	Directional sign and other visual element data	.shp, .dbf, .shx, .prj
Traffic Sign (Line)	Pangyo_B2_SURFSIGN_LINE_3D	Linear traffic sign data	.shp, .dbf, .shx, .prj
Traffic Sign (Plane)	Pangyo_B2_SURFSIGN_PLANE_3D	Area-type traffic sign data	.shp, .dbf, .shx, .prj
Node	Pangyo_C1_NODE_3D	Node-to-link connection information	.shp, .dbf, .shx, .prj

**Table 3:** Comparison of Geohash levels in terms of block size and RTT.

Geohash Level	Average Block Size	Average RTT
Level 4	2.5 GB	6.0 s
Level 5	700 MB	3.0 s
Level 6	230 MB	1.6 s

**Table 4:** Schema of HD Map Data Tables.

Table Name	Column Name
Pangyo_A1_LANE_3D	Id, Geom, Lanecode, Geohash6
pangyo_A2_STOP_3D	Id, Geom, Geohash6
Pangyo_A3_LINK_3D	Id, Geom, Linkid, Fromnode, Tonode, Lane, Route_id, Geohash6
Pangyo_B2_SURFSIGN_DIRECTION_3D	Id, Geom, Signtype, Geohash6
Pangyo_B2_SURFSIGN_LINE_3D	Id, Geom, Signtype, Geohash6
Pangyo_B2_SURFSIGN_PLANE_3D	Id, Geom, Signtype, Geohash6
Pangyo_C1_NODE_3D	Id, Geom, Nodeid, Nodetype, Geohash6

**Table 5:** Continuity of Boundary Roads Between Adjacent Geohash Blocks (“↔” denotes adjacency).

Geohash Pair	Number of Boundary Roads	Number of Common Roads	Continuity (%)
wydksx ↔ wydksr	7070	70	100
wydksr ↔ wydksq	59	59	100
wydksq ↔ wydksw	12	12	100
wydksw ↔ wydksx	113	113	100

## Data Availability

Data are available upon request from the authors.
